# Hematological Markers as Predictors of Attack and Remission Phases in Pediatric Familial Mediterranean Fever

**DOI:** 10.3390/jcm15103857

**Published:** 2026-05-17

**Authors:** Besim Hacioglu, Ferah Sonmez

**Affiliations:** 1 Republic of Turkey Ministry of Health, 47000 Siirt, Turkey; 2 Department of Pediatric Nephrology, Faculty of Medicine, Bezmialem University, 21000 Istanbul, Turkey; ferah.sonmez@bezmialem.edu.tr

**Keywords:** biomarker, disease activity markers, hematological biomarkers, pediatric rheumatology, platelet indices

## Abstract

**Background:** The study aimed to evaluate whether routine hematological parameters can differentiate attack and remission phases in children with Familial Mediterranean Fever (FMF) and to identify accessible biomarkers for disease activity monitoring. **Methods**: This retrospective study included pediatric FMF patients diagnosed according to Tel Hashomer, Livneh, or Yalçınkaya criteria at Aydın Adnan Menderes University between April 2000 and April 2020. Patients with complete blood count data available for both attack and remission phases were analyzed. Hematological parameters included leukocyte, neutrophil, lymphocyte, and platelet counts, plateletcrit (PCT), mean platelet volume (MPV), and platelet distribution width (PDW). Paired comparisons were performed using appropriate statistical tests. Independent predictors of disease phase were identified using a multivariate mixed-effects logistic regression model. **Results**: Eighty-seven patients (mean age at diagnosis 7.1 ± 3.6 years) were included. Neutrophil counts were significantly higher during attacks than remission (*p* < 0.001). Although lymphocyte counts were not significant in univariate analysis, higher lymphocyte concentration was independently associated with remission (OR = 1.71, 95% CI: 1.19–2.45). PDW was significantly higher during remission and independently predicted remission status (OR = 1.92, 95% CI: 1.30–2.85). PCT and MPV were not significant predictors. **Conclusions:** Neutrophil count, lymphocyte count, and PDW may represent potential and accessible markers associated with FMF attack and remission phases in pediatric patients.

## 1. Introduction

C Familial Mediterranean Fever (FMF) is a hereditary autoinflammatory disease characterized by recurrent episodes of fever, abdominal discomfort, joint pain, and occasionally skin rashes [1,2,3]. FMF typically begins before the age of five in two-thirds of patients. The attacks are generally self-limiting, lasting 1–3 days. However, even during asymptomatic periods, persistent subclinical inflammation can occur, contributing to the development of various comorbidities, such as anemia, cardiovascular disease, and amyloidosis [4].

Despite advances in genetic testing, FMF diagnosis relies heavily on clinical symptoms and genetic analysis, underscoring the need for additional biomarkers to reflect disease activity and facilitate more precise monitoring accurately. In contrast to previous research [5,6,7,8,9,10,11,12,13,14,15,16,17,18,19] that often compared FMF patients to healthy controls or those in different disease phases, this study focuses on intra-patient variability.

This study aims to assess whether specific routine hematological parameters may help distinguish between the attack and remission phases of FMF in children, identifying reliable biomarkers that can be integrated into clinical practice for improved diagnostic precision and personalized treatment.

## 2. Materials and Methods

### 2.1. Study Subjects

A retrospective review was conducted on pediatric patients diagnosed with FMF based on Tel Hashomer, Livneh, or Yalçınkaya criteria at Aydın Adnan Menderes University Faculty of Medicine, Department of Pediatric Nephrology, between 1 April 2000 and 1 April 2020. Patients were included if they had complete blood count data available for both attack and remission phases. The attack phase was defined as the presence of clinically active FMF symptoms (fever, abdominal pain, arthritis, serositis) requiring clinical evaluation, while the remission phase was defined as a clinically asymptomatic period. Patients for whom complete data could not be obtained and those who refused to participate were excluded from the study.

### 2.2. Study Methods

#### Demographic, Clinical, and Laboratory Data

Age at diagnosis, sex, FMF genetics, clinical data, attack phase, treatment, response to treatment, and complete blood count parameters were extracted from patient records. The researchers assessed the appropriateness of the diagnostic criteria for each patient. Elevated levels of any single acute-phase reactant, including white blood cell count, C-reactive protein, fibrinogen, erythrocyte sedimentation rate, and Serum Amyloid A (SAA) during the remission phase, were defined as subclinical inflammation. Blood samples taken during the active phase were collected at the time of clinical presentation, when active symptoms were present. Remission samples, on the other hand, were collected during routine outpatient follow-up visits when patients were clinically asymptomatic.

In this study, the hematological parameters analyzed were as follows: leukocyte count, neutrophil count, lymphocyte count, platelet count, plateletcrit (PCT), mean platelet volume (MPV), and platelet distribution width (PDW). Leukocyte, neutrophil, lymphocyte, and platelet counts are presented in thousands per microliter (×10^3^/µL). PCT is reported as a percentage (%) of the volume of blood it occupies. The MPV was measured in femtoliters (fL), and the PDW was expressed in percentage (%) to reflect variation in platelet size.

### 2.3. Statistical Analysis

Normality of the paired differences between attack and remission values was assessed using the Shapiro–Wilk test. Leukocyte count, neutrophil count, platelet count, and MPV were analyzed with the paired *t* test, while lymphocyte count, PCT, and PDW were analyzed with the Wilcoxon signed-rank test because their paired differences were not normally distributed.

A mixed-effects logistic regression model was constructed to identify independent predictors of disease phase. The unit of analysis was the phase-specific observation, with each patient contributing two observations, one during the attack phase and one during the remission phase. Disease phase was entered as the dependent variable, and a patient-specific random intercept was included to account for within-patient pairing of attack and remission measurements. Hematological parameters were entered as fixed effects. The model was adjusted for age at diagnosis, sex, homozygous M694V mutation status, attack duration, and attack intensity. Attack duration and attack intensity were incorporated as categorical covariates according to the categories presented. Before creating the model, the researchers examined highly correlated variables (Pearson correlation coefficient >0.5) to avoid multicollinearity [20]. Among these correlated variables, selections were made to retain only the most relevant predictors for the analysis. The odds ratio (OR) and its 95% confidence intervals for each parameter in the final model were reported.

Model performance results, including AUC (Area Under the Curve), sensitivity, specificity, positive predictive value (PPV), and negative predictive value (NPV), are reported. Analysis was performed using R 4.2.0, and *p* < 0.05 was considered statistically significant.

The Aydın Adnan Menderes University Non-Interventional Clinical Research Ethics Committee (date: 18 August 2020, number: E.42341) approved the study.

## 3. Results

**Figure 1:**
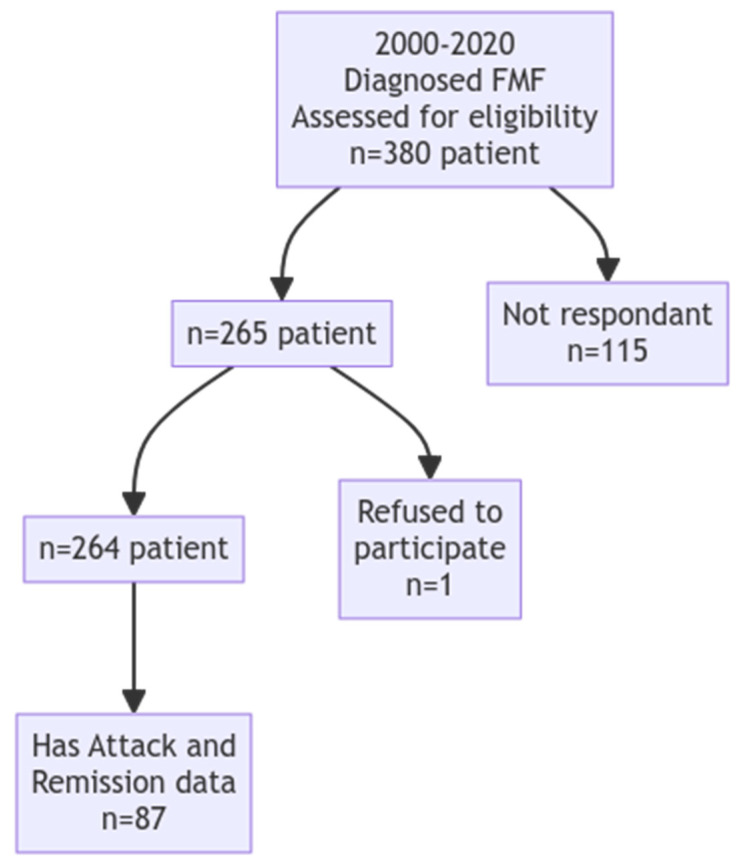
Flowchart.

A total of 380 patients were assessed for eligibility, of which 265 met the inclusion criteria. One patient refused to participate. The final analysis was conducted on 87 patients with complete data for both the attack and remission phases. A flowchart illustrates the patient selection process (Figure 1).

Table 1 summarizes patient characteristics. The mean age at diagnosis was 7.1 ± 3.6, and the current mean age is 11.2 ± 4.2 years. 36.8% of patients were male. Additional diseases co-occurring with FMF were found in 9.2% of patients.

Regarding phenotype, 97.7% (*n* = 85) of the patients experienced repeated inflammatory attacks (Type 1), 1.1% (*n* = 1) had amyloidosis (Type 2), and 1.1% (*n* = 1) were asymptomatic but carried mutations (Type 3).

MEFV genetic analysis was available for 86 patients. Among these, 79 patients (91.9%) had at least one detected MEFV variant, while 7 patients (8.1%) had no detected variant. Regarding zygosity, 3 patients (3.5%) had compound heterozygous or double variants, 14 patients (16.3%) were homozygous, and 62 patients (72.1%) were heterozygous. Pathogenic variants were identified in 61 patients (70.1%), whereas 26 patients (29.9%) had benign variants or variants of uncertain significance. A family history of FMF was present in 41.4% of patients. The prevalence of subclinical inflammation was 41.4%.

Table 2 details the characteristics of FMF attacks observed in the study population. The attacks experienced by 87.4% (*n* = 76) of the patients lasted between 3 and 72 h. Most attacks (81.6%) were severe and needed (88.5%, *n* = 77) colchicine treatment. Most patients, comprising 89.6%, exhibited a favorable response to colchicine treatment, while a smaller proportion, 7.8%, experienced a partial response. The most prevalent (51.9%) treatment regimen was colchicine at a dosage of 1 mg per day. Additionally, three patients required IL-1 inhibitor therapy, with 2 exhibiting a complete response. The FMF50 score was considered appropriate in 74 (97.4%) patients.

**Figure 2:**
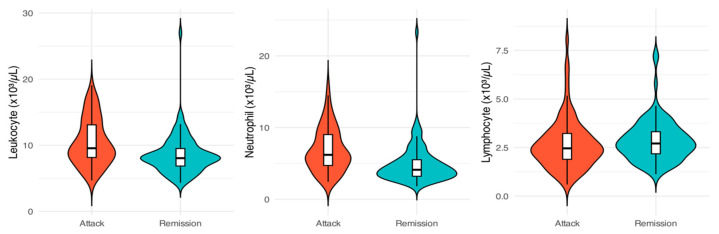
Comparison of Leukocyte, Neutrophil, and Lymphocyte Counts During FMF Attack and Remission Phases.

**Figure 3:**
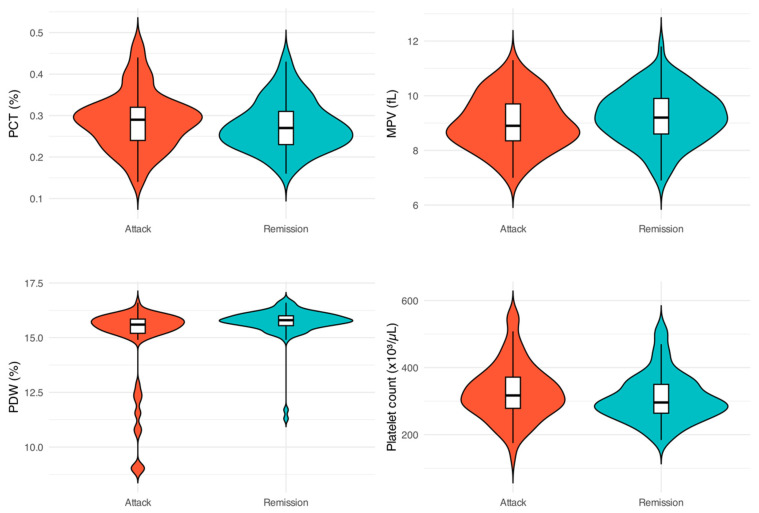
Comparison of Platelet Parameters During FMF Attack and Remission Phases.

The hematological parameters of patients during the attack and remission phases were compared using paired tests selected according to the normality of paired differences and the results are presented in Table 3. Figure 2 and Figure 3 further illustrate the differences in these parameters between the two phases.

Leukocyte and neutrophil counts were both significantly higher during the attack phase compared with the remission phase (leukocyte: 10.45 ± 3.48 × 10^3^/µL vs. 8.55 ± 3.01 × 10^3^/µL, *p* < 0.001; neutrophil: 6.86 ± 3.03 × 10^3^/µL vs. 4.90 ± 2.80 × 10^3^/µL, *p* < 0.001). There was no statistically significant difference in lymphocyte counts between the two phases (*p* = 0.181). Platelet counts were significantly higher during attacks (327.32 ± 83.86 × 10^3^/µL) compared to remission (308.51 ± 70.75 × 10^3^/µL, *p* = 0.011). PCT values showed a non-significant reduction during remission (*p* = 0.068). MPV was lower during attacks (9.02 ± 0.98 fL) compared to remission (9.24 ± 0.95 fL, *p* = 0.001). Conversely, PDW was significantly higher during the remission phase (15.70 ± 0.74%) compared to the attack phase (14.82 ± 1.94%, *p* < 0.001).

**Figure 4:**
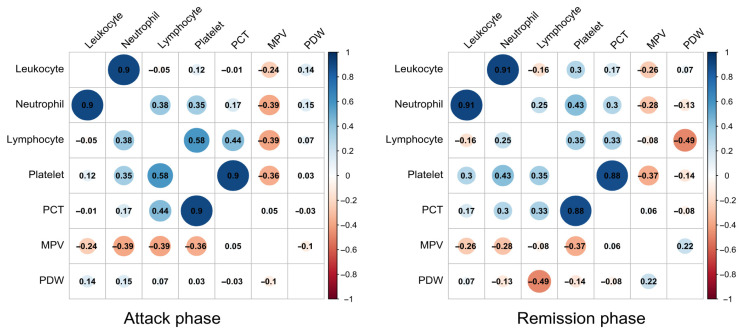
Correlation Matrices of Hematological Parameters in Patients During Attack and Remission Phases.

The correlation matrices (Figure 4) highlight several strong correlations among hematological parameters during both FMF attack and remission phases. During the attack phase, notable positive correlations were observed between leukocyte and neutrophil counts (r = 0.90), lymphocyte and platelet counts (r = 0.58), as well as platelet count and PCT (r = 0.90). In the remission phase, similarly strong correlations were found between leukocyte and neutrophil counts (r = 0.91), platelet count, and PCT (r = 0.88). Additionally, lymphocyte count was moderately correlated with both platelet count (r = 0.58) and PCT (r = 0.44) during the attack phase; however, these correlations were slightly weaker in the remission phase.

Differences in hematological parameters between the attack and remission phases were examined using a multivariate mixed-effects logistic regression model (Table 4). The model was adjusted for age at diagnosis, sex, presence of homozygous M694V mutation, attack duration, and attack intensity.

A lower neutrophil count was associated with a higher likelihood of being in the remission phase (OR = 0.77, 95% CI: [0.67–0.89]). Conversely, a higher lymphocyte count (OR = 1.71, 95% CI: [1.19–2.45]) was significantly associated with a higher likelihood of being in the remission phase. Higher PDW was also significantly associated with a higher likelihood of being in the remission phase (OR = 1.92, 95% CI: [1.30–2.85]).

Other variables, including PCT and MPV, did not demonstrate significant predictive value (PCT: OR = 0.01, 95% CI: [0.00–4.94]; MPV: OR = 1.27, 95% CI: [0.83–1.94]).

The model exhibited an AUC of 0.78 (95% CI: 0.70–0.84) and an AIC of 221.07. The performance metrics were 87.35% sensitivity, 62.06% specificity, 83.07% NPV, and 69.72% PPV.

## 4. Discussion

This study evaluated whether routine hematological parameters could effectively distinguish between the phases of FMF attack and remission in pediatric patients. Using a multivariate mixed-effects logistic regression model adjusted for potential confounders, we identified neutrophil count, lymphocyte count, and PDW as significant independent predictors of disease phase.

Our findings indicate that elevated neutrophil counts, key components of the innate immune response, are associated with acute inflammation during FMF attacks. In contrast, higher lymphocyte counts, reflecting adaptive immunity and immune homeostasis, are significantly linked to the remission phase, as revealed by multivariate analysis, despite non-significant differences in univariate analysis. This observation is consistent with several studies. For instance, Gönüllü et al. found elevated neutrophils in the attack group without differences in lymphocytes using a methodology similar to ours in adult patients [21]. Aygün et al. and Doğruel et al. similarly reported higher leukocyte counts in the attack group without differentiating between neutrophils and lymphocytes [22,23]. Ahsen et al. introduced the neutrophil-to-lymphocyte ratio as a new criterion in FMF. They observed differences in neutrophil counts between FMF patients and controls but not in lymphocytes [6]. Başaran et al. reported increased neutrophilia in the attack group and increased lymphocytes in the remission group, findings that align with ours [8]. These outcomes suggest that neutrophil count, lymphocyte count, and derived inflammatory indices such as NLR may have potential value in reflecting disease activity in FMF patients.

In addition to neutrophil-lymphocyte counts, PDW also emerged as a significant predictor in our study, with higher values observed during remission. Increased PDW might reflect more significant heterogeneity in platelet size due to the normalization of platelet production following consumption during acute inflammation. However, studies have reported that PDW is not an important marker. For example, Gönüllü et al. did not detect differences in PDW in the follow-up data of the same individuals, similar to our study [21]. Similarly, Uluca et al. found no significant differences in PDW among attack, remission, and control groups, and Kazan et al. did not observe substantial PDW differences between FMF patients in remission and controls or according to disease severity [17,24]. These discrepancies may result from variations in study design, patient demographics, and methodological approaches, such as the timing of blood sample collection, adherence to colchicine therapy, and the presence of comorbidities. In contrast to other studies, a mixed-effects logistic regression model suggests that PDW may play a role in monitoring FMF activity.

PCT was another research variable in our study. High PCT values may indicate increased platelet activity in FMF. In our research, PCT showed a high correlation with platelet count during the attack phase (r = 0.90). We included PCT in the final model, considering the correlation between platelets and lymphocytes (attack phase, r = 0.58). Although studies documenting platelet differences in FMF are frequent [7,11,15], studies on PCT are limited. In the only other study in the literature in this area, conducted by Kazan et al., no differences in PCT were found when comparing adult FMF patients in remission with a control group [24]. These findings highlight the inconsistent role of PCT as a biomarker in FMF across different studies. PCT provides quantitative information about the total platelet mass, incorporating count and size. It can also detect alterations in platelet mass that might not be evident through platelet count alone, offering additional diagnostic insight, especially in nuanced clinical scenarios. Given the limited and inconsistent findings, future studies should investigate the role of PCT in conjunction with other hematological markers and across larger, diverse populations to determine its potential utility as a biomarker for FMF disease activity.

MPV was another variable examined in our study. Although univariate analyses indicated higher MPV during the remission phase, this difference was not observed in the multivariate analysis. MPV is a significant indicator of platelet activity and inflammation. Consistent with the studies by Uluca et al. and Gönüllü et al., which reported no differences in MPV values between FMF attack and remission periods, our research also found no significant difference between these two phases [17,21]. In studies comparing FMF patients in remission with controls, Dinçer et al. found MPV was lower in FMF patients [25].

In contrast, Aygün et al. found no differences in MPV between FMF patients in remission and controls [22]. This finding suggests that MPV alone may not be a sufficient marker for monitoring disease activity in FMF patients. In contrast to other studies, our study, which did not include a control group, supports that MPV does not provide a consistent indicator across different phases of FMF [7,14,15,23].

In our study, subclinical inflammation was identified in 41.4% of pediatric FMF patients, underscoring its critical role in the disease’s progression and the potential development of severe complications such as amyloidosis. Recent research has increasingly focused on disease severity and subclinical inflammation in FMF. Tezcan et al. associated a poor prognosis with a decreased mean erythrocyte hemoglobin concentration, elevated neutrophil and monocyte counts, and an increased NLR [26]. Ocak et al. identified Pan-Immune-Inflammation Value and Red Cell Distribution Width (RDW) as significant markers for predicting disease severity [27]. At the same time, Güngörer and Arslan reported no relationship between disease severity and RDW, Red Cell Distribution Width-Platelet Ratio, NLR, and Platelet-Lymphocyte Ratio using the International Severity Score of FMF [28]. Parmaksız and Noyan found RDW a helpful marker for subclinical inflammation, particularly in patients with specific MEFV mutations [29]. Additionally, Çakan et al. proposed SAA as the most potential marker for defining subclinical inflammation [30]. These findings underscore the complexity of utilizing hematological markers in FMF, suggesting that a combination of multiple biomarkers may be necessary for accurate disease assessment and management.

This study has several limitations. The retrospective design may introduce selection bias, as only patients with complete data for both the attack and remission phases were included, thereby reducing the sample size. The absence of a healthy control group limits our ability to compare hematological parameters against baseline values in the general pediatric population. Additionally, most patients (77/87) received colchicine treatment, which could modulate hematological parameters and mask the natural variations associated with disease activity. The study’s extended time frame may also introduce variability due to changes in diagnostic criteria, laboratory techniques, and treatment protocols that have occurred over the years. Due to the limited sample size, a separate sensitivity analysis excluding patients with subclinical inflammation was not performed. Due to the retrospective design of the study, the timing and intervals of blood sample collection could not be fully standardized.

## 5. Conclusions

In conclusion, our study suggests that routine hematological parameters, specifically neutrophil count, lymphocyte count, and PDW, may help distinguish between the attack and remission phases of FMF in pediatric patients. The differences between our findings suggest that routine hematological parameters, particularly neutrophil count, lymphocyte count, and PDW, may be associated with FMF attack and remission phases in pediatric patients. Further studies using standardized methodologies are needed to clarify the roles of these hematological parameters in FMF and determine their potential utility in clinical practice.

## Figures and Tables

**Table 1:** Demographic Characteristics of Pediatric FMF Patients (*n* = 87).

Characteristic	N (%, Percent)
Age at diagnosis (years) *	7.1 (3.6)
Sex	
Male	32 (36.8%)
Female	55 (63.2%)
Comorbidities	
Present	8 (9.2%)
None	79 (90.8%)
Phenotype	
Repeated inflammatory attacks (Type 1)	85 (97.7%)
Amyloidosis (Type 2)	1 (1.1%)
No symptoms but mutation (Type 3)	1 (1.1%)
MEFV mutation status **	
Compound heterozygous or double variant	3 (3.5%)
Homozygous	14 (16.3%)
Heterozygous	62 (72.1%)
None	7 (8.1%)
Mutation significance	
Pathogenic	61 (70.1%)
Benign or uncertain significance	26 (29.9%)
MEFV genotype	
M694V homozygous	10 (11.5%)
Other	77 (88.5%)
FMF family history	
Present	36 (41.4%)
Absent	51 (58.6%)
Subclinical inflammation	
Present	36 (41.4%)
Absent	51 (58.6%)

* Mean (Standard deviation). ** MEFV genetic analysis was available for 86 patients.

**Table 2:** Clinical Characteristics of FMF Attack Phase.

Characteristic	N (%, Percent)	Total N
Attack duration		87
3–72 h	76 (87.4%)	
>72 h	11 (12.6%)	
Attack intensity		87
Severe	71 (81.6%)	
Mild	16 (18.4%)	
Colchicine dose (mg/day)		77
0.5 mg/day	22 (28.6%)	
1 mg/day	40 (51.9%)	
1.5 mg/day	8 (10.4%)	
2 mg/day	7 (9.1%)	
Colchicine response		77
No response	2 (2.6%)	
Partial response	6 (7.8%)	
Response	69 (89.6%)	
IL-1 Use		77
Yes	3 (3.9%)	
No	74 (96.1%)	
IL-1 response		3
Complete response	2 (66.7%)	
No response	1 (33.3%)	
FMF50 score		76
Appropriate	74 (97.4%)	
Inappropriate	2 (2.6%)	

**Table 3:** Comparison of Hematological Parameters During FMF Attack and Remission Phases (n = 87).

	Attack	Remission	*p*-Value
Leukocyte (×10^3^/μL)	10.45 (3.48)	8.55 (3.01)	<0.001
Neutrophil (×10^3^/μL)	6.86 (3.03)	4.90 (2.80)	<0.001
Lymphocyte (×10^3^/μL)	2.68 (1.32)	2.85 (1.09)	0.181
Platelet (×10^3^/μL)	327.32 (83.86)	308.51 (70.75)	0.011
PCT (%)	0.29 (0.07)	0.28 (0.06)	0.068
MPV (fL)	9.02 (0.98)	9.24 (0.95)	0.001
PDW (%)	14.82 (1.94)	15.70 (0.74)	<0.001

Values are presented as mean ± standard deviation. Normality of paired differences between attack and remission values was assessed using the Shapiro–Wilk test. Leukocyte count, neutrophil count, platelet count, and MPV were analyzed using the paired *t* test; lymphocyte count, PCT, and PDW were analyzed using the Wilcoxon signed-rank test.

**Table 4:** Mixed-Effect Logistic Regression Analyses Investigating the Impact of Variables on FMF Attack and Remission.

Predictors	Multivariate OR	95% CI	*p*
Neutrophil count	0.77	0.67–0.89	<0.001
Lymphocyte count	1.71	1.19–2.45	0.004
PCT	0.01	0.00–4.94	0.139
MPV	1.27	0.83–1.94	0.264
PDW	1.92	1.30–2.85	0.001

OR: odds ratio; CI: confidence interval; NPV: negative predictive value; PPV: positive predictive value. The model included a patient-specific random intercept to account for paired observations within individuals and was adjusted for age at diagnosis, sex, homozygous M694V mutation status, attack duration, and attack intensity. Disease phase was coded as remission versus attack; therefore, odds ratios greater than 1 indicate higher odds of remission. Model performance was as follows: area under the curve, 0.78 (95% CI: 0.70 to 0.84); AIC, 221.07; log-likelihood, −98.53. At the Youden index threshold of 42.76, sensitivity was 87.35%, specificity was 62.06%, NPV was 83.07%, and PPV was 69.72%.

## Data Availability

The datasets used and/or analyzed during the current study are available from the corresponding author on reasonable request.
